# Syndrome d'interruption de la tige pituitaire à révélation tardive

**DOI:** 10.11604/pamj.2016.23.108.8801

**Published:** 2016-03-16

**Authors:** Héla Marmouch, Samah Graja, Sondes Arfa, Fadia Boubaker, Ines Khochtali

**Affiliations:** 1 Service de Médecine Interne-Endocrinologie, Hôpital Universitaire Fattouma Bourguiba à Monastir,Tunisie

**Keywords:** Syndrome d′interruption de la tige pituitaire, hypopituitarisme, retard de croissance, impubérisme, Pituitary stalk interruption syndrome (PSIS), hypopituitarism, growth retardation, impuberism

## Abstract

Le syndrome d'interruption de la tige pituitaire est une cause assez fréquente de déficit en hormone de croissance et d'hypopituitarisme souvent révélé pendant la période néonatale et l'enfance. Cette observation illustre les particularités d'une révélation tardive de ce syndrome. Il s'agit d'une patiente âgée de 17ans hospitalisée pour aménorrhée primaire et impubérisme. Elle n'a pas d'antécédent d'incident néonatal. L'examen clinique révèle un retard de croissance sévère. L'hypophysiogramme a montré un hypopituitarisme complet sans diabète insipide. L'imagerie par résonnance magnétique a montré une interruption de la tige pituitaire avec une post hypophyse ectopique. Une malformation rénale a été objectivée, ce qui est en faveur d'une origine congénitale malformative de ce syndrome. Une substitution hormonale a été administrée à cette patiente. Cette forme clinique tardive souligne la nécessité de diagnostic précoce d'impubérisme et/ou de retard de croissance révélant une pathologie à potentiel de gravité important.

## Abstract (en)

Pituitary stalk interruption syndrome is a fairly common cause of the deficiency of Growth Hormone and hypopituitarism often revealed in the neonatal period and childhood. This observation illustrates the peculiarities of a late clinical onset of this syndrome. We report a case of a 17-year-old patient hospitalized for primary amenorrhoea and impuberism. She had no history of neonatal incident. Clinical examination revealed severe growth retardation Hypophysiogramme showed complete hypopituitarism without diabetes insipidus. Magnetic resonance imaging revealed pituitary stalk interruption and an ectopic posterior pituitary gland. Kidney malformation was objectified, which is in favour of a congenital malformative origin of this syndrome. Hormone replacement was administered to this patient. This late-onset form emphasizes the need for early diagnosis of impuberism and/or stunting, revealing a potentially very serious pathology.

## Introduction

Les retards de croissance sont diagnostiqués précocement dans les pays ayant un système de santé développé et généralisé. Les déficits hypophysaires sont classiquement révélés par un retard de croissance harmonieux et restent sans doute sous-estimés dans les régions défavorisées. L'avènement de l'imagerie par résonance magnétique (IRM) a permis de mieux étayer le diagnostic étiologique dans ce contexte. Des anomalies morphologiques hypothalamo-hypophysaires ont été décrite pour la première fois en 1987 par Fujisawa et al [1]. Il s'agit du syndrome d'interruption de la tige pituitaire (SITP) qui correspond à une définition anatomique. Il se traduit par des anomalies morphologiques mises en évidence par l'IRM à savoir une tige pituitaire grêle, une hypoplasie antéhypophysaire et une posthypophyse ectopique. L’âge de découverte est généralement précoce, en période néonatale ou pendant l'enfance et les circonstances de diagnostic sont celles d'un hypopituitarisme isolé ou multiple. Nous rapportons cette observation qui souligne les particularités d'une révélation tardive de ce syndrome.

## Patient et observation

Une patiente âgée de 17 ans consulte pour aménorrhée primaire. Elle a un bas niveau socio-économique et demeurant dans une région du Sud-ouest de la Tunisie. L′examen a trouvé un retard staturo-pondéral sévère et un impubérisme. L′interrogatoire n′a révélé aucun antécédent de souffrance fœtale, ni incident néonatal, ni traumatisme crânien, ni cas similaire dans la famille. Le bilan hormonal avait révélé un hypopituitarisme complet. Un retard scolaire était noté. Le quotient intellectuel était estimé à 70%.

A l'examen, on note une taille à 140 cm (-3S), un poids de 42 kg, une voix infantile, une pilosité axillaire absente, une pilosité pubienne minime, soit un stade I de Tanner. Il n'y a pas de syndrome malformatif associé et on note une légère adiposité abdominale. L'exploration biologique initiale n'a pas montré de stigmates de malabsorption ni de parasitose. La radiographie pulmonaire et l’électrocardiogramme sont normaux. L’âge osseux est de 11 ans.

**Figure 1:**
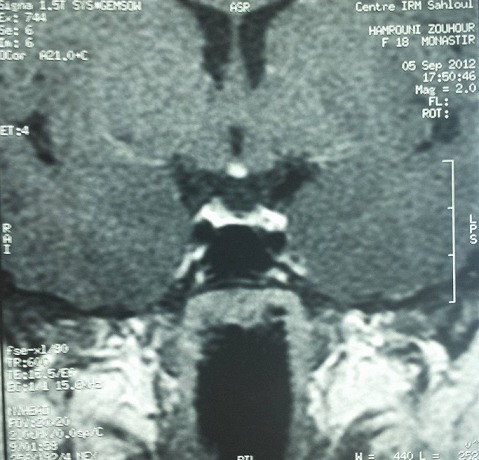
Coupe frontale de l'IRM hypothalamo-hypophysaire: interruption de la tige pituitaire

**Figure 2:**
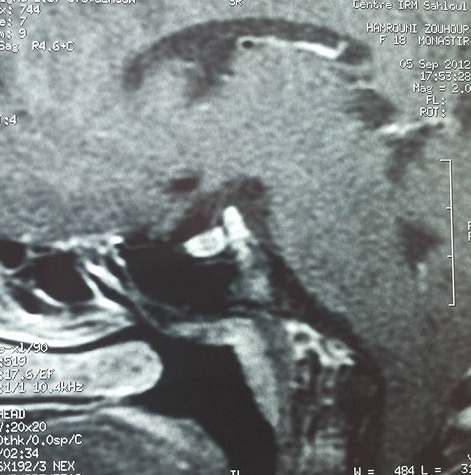
Coupe sagittale d'IRM Hypothalamo-hypophysaire: post hypophyse ectopique

Le bilan hormonal a montré un œstradiol bas à 6 ng/l, LH à0.3 UI/l et FSH à 0.8 IU/l diminuées avec une prolactinémie basse à 115 mUI/l, des IGF1 bas à 46 ng/ml (normal: 127 - 903 ng/ml), une TSH basse à 0.02 mUI/l (normal: 0.5-4.5) avec FT4 très basses à 3 pmol/l (normal: 9-18) et une cortisolémie de base effondrée à 20 ng/ml. Il s'agit d'un hypopituitarisme complet. L'IRM hypothalamo-hypophysaire, en coupe frontale, a objectivé une glande antéhypophysaire en place hypoplasique, de rehaussement homogène avec une tige pituitaire est mal individualisée (Figure 1). La post hypophyse est en situation ectopique haut située dans l’éminence médiane sous l'hypothalamus; elle est de signal normal à l'IRM en coupe sagittale (Figure 2).

Une malformation rénale a été découverte à l’échographie. Le caryotype de cette patiente est 46 XX. L'ostéodensitométrie a retrouvé une ostéoporose sévère. Une hormonothérapie substitutive à base d′hydrocortisone, thyroxine, et hormone de croissance (GH) a été instaurée et secondairement une introduction d'oestrogènothérapie. La patiente sera suivie régulièrement pour évaluation clinico-biologique. L’évolution sous traitement est marquée par un gain de taille de 10 cm en 4 ans atteignant ainsi une taille de 150 cm.

## Discussion

L'insuffisance anté-hypophysaire de l'enfant est d'origine multifactorielle (malformative, génétique, traumatique, tumorale…). Une entité particulière est individualisable: le syndrome d'interruption de la tige pituitaire (SITP) défini par des anomalies morphologiques mises en évidence à l'IRM: tige pituitaire non visible, hypoplasie hypophysaire et post hypophysaire ectopique [1]. Ce syndrome constitue ainsi une étiologie du déficit hypophysaire et se traduit sur le plan clinico-biologique par un déficit hypophysaire unique ou multiple [2, 3]. Dans notre cas, il s'agit d'un retard statural et impubérisme découverts chez une adolescente de 17 ans. Quelques publications ont rapporté un âge post pubertaire au diagnostic ou l'axe gonadotrope est conservé [1, 3, 4]. Pour notre patiente, le déficit hormonal a intéressé tous les axes hypophysaires ce qui explique le tableau clinique. L′IRM met en évidence une tige pituitaire non individualisable, d′une hypoplasie de l′anté-hypophyse et d′une posthypophyse en position ectopique ce qui définit le SITP. L′absence de diabète insipide, initial et après supplémentation des axes hypophysaires surtout corticotrope, témoigne du caractère fonctionnel de la posthypophyse confirmé par la conservation du signal de la post hypophyse ectopique à l'IRM. En effet, la plupart des études semblent montrer une bonne corrélation entre la présence de l′hypersignal et le caractère fonctionnel de la post-hypophyse [5]. Deux hypothèses étio-pathogéniques ont été proposées pour le SITP [1]: (a) la première est la souffrance périnatale avec une fréquence variant de 50 à 60% des cas [1]. La présence d'un syndrome malformatif (tel qu'une imperforation anale, une atteinte ophtalmologique ou du système nerveux central… ou de cas familiaux fait suspecter un début anténatal et probablement génétique de la maladie. Ainsi, certains auteurs suggèrent que la souffrance périnatale n'est en fait qu'un élément du tableau du déficit endocrinien [1, 6]. En fait les formes malformatives peuvent être diagnostiquées après la période néonatale; (b) la deuxième hypothèse est un mécanisme traumatique et vasculaire; les formes traumatiques peuvent être périnatales (souffrance fœtale, anoxie, naissance par siège) ou post natales (traumatisme crânien). Elles peuvent être soit purement mécaniques par étirement ou par section de la tige pituitaire par le diaphragme sellaire, soit vasculaire par anoxie, ischémie ou hémorragie [1].

L′hypothèse traumatique (étirement ou section de tige avec ou sans participation vasculaire ischémique) a laissé place à la théorie malformative anténatale, en raison de malformation rénale associée, malgré que l′âge au moment du diagnostic de ces anomalies est habituellement inférieur à 3 ans dans la plupart des cas rapportés [1, 3, 6]. Les cas néonataux sont souvent diagnostiqués devant des hypoglycémies sévères, associés ou non à un syndrome malformatif [6]. Chez les enfants plus âgés, les circonstances sont soit un ralentissement de la vitesse de croissance, soit plus rarement un diabète insipide.

Des mutations des gènes codant pour les facteurs de transcription impliqués dans l'ontogenèse antéhypophysaire ont été évoquées. Certains auteurs suggèrent que la souffrance périnatale n'est en fait qu'un élément du tableau du déficit endocrinien. En fait les formes malformatives peuvent être diagnostiquées après la période néonatale et parfois même à l’âge d'adolescence comme le cas de notre patiente. Une origine anténatale génétique est plutôt évoquée [3, 6]. II existe donc une certaine évolutivité, les déficits endocriniens devenant, avec le temps, plus intenses et multiples, les observations les plus récentes ayant les déficits les moins complets et les plus isolés. Cette évolutivité n′est pas soulignée dans les publications, mais les formes cliniques différentes correspondent probablement à des stades évolutifs différents [7]. Hasegawa et al décrivent le cas d′un garçon qui présente un retard statural sans déficit à 5 ans, puis un déficit partiel de GH à 13 ans [8].

Le tableau clinique insidieux bien toléré avec une atteinte endocrine progressive dans ce contexte impose une surveillance régulière d'autant qu'avec le temps les déficits deviennent intenses et multiples comme illustre le cas de notre patiente. Chez notre patiente, un délai diagnostic long est à l'origine d'un retard statural sévère. Ce délai a été favorisé par de mauvaises conditions socio-économiques et culturelles et par l'absence de symptomatologie aiguë.

## Conclusion

Le syndrome d'interruption de la tige pituitaire est habituellement révélé en période néonatale ou pendant la petite enfance; les cas à diagnostic tardif sont l'apanage des formes bien tolérées mais leur pronostic statural péjoratif doit inciter au dépistage précoce des retards staturo-pubertaires et à leur exploration rigoureuse.
